# Intratumoural injection of absolute alcohol in carcinoma of gastroesophageal junction for palliation of dysphagia

**DOI:** 10.3332/ecancer.2014.395

**Published:** 2014-02-03

**Authors:** Vishnu Prasad Nelamangala Ramakrishnaiah, J Ramkumar, Dinker Pai

**Affiliations:** Department of Surgery, Jawaharlal Institute of Postgraduate Medical Education and Research (JIPMER), Puducherry 605006, India

**Keywords:** oesophageal cancer, palliative therapy, endoluminal therapy

## Abstract

**Background:**

Ethanol-induced tumour necrosis (ETN) is a simple, readily available palliative treatment for patients with inoperable carcinoma of the oesophagus with poor performance status. In India, capital outlay needed for stenting or laser therapy is out of reach. Hence, we took up this study to calculate the effect of intratumoral injection of absolute alcohol in palliation of dysphagia due to carcinoma of the oesophagogastric junction and to monitor the improvement in quality of life (QOL).

**Methods:**

A total of 16 patients with a mean age of 56.2 ± 7.5 years with dysphagia due to unresectable malignant oesophageal strictures involving the oesophagogastric junction were included in the study. Six to ten cubic centimetres of absolute alcohol in 1 cc aliquots was injected circumferentially into the tumour at the point of luminal obstruction using disposable sclerosing needles (23G). During each follow up dysphagia grade, QOL score and complications, if any, were noted.

**Results:**

The mean alcohol injected per session was 6.9 ± 1.8 cc. The mean dysphagia grade improved from 5.5 ± 0.5 to 2.5 ± 1.1 before and after alcohol injection, respectively (*p* < 0.001). The time taken for recurrence of dysphagia ranged from 14 to 80 days with a median of 28 days. The mean QOL score assessed by modified EORTC questionnaire improved from a mean of 63.6 ± 6.9 to 92.6 ± 13.9 (*p* < 0.001). The dysphagia free survival ranged from 23 to 175 days with a mean of 71.2 days. Complications included oesophageal perforation in one patient and death in one patient.

**Conclusion:**

The endoscopic intratumoral injection of absolute alcohol significantly improves dysphagia and QOL. It is inexpensive and easy to perform.

## Background

The majority of patients with oesophageal carcinoma have inoperable disease, and without treatment, the ability to swallow deteriorates rapidly, with a dramatic effect on quality of life (QOL) [1]. The predominant symptom is progressive dysphagia, which occurs once there has been at least a 60% reduction in lumen diameter [2, 3]. Thus, the primary therapeutic objective is palliation of dysphagia with minimum morbidity, mortality, or need for hospitalisation.

Surgical palliation is rarely indicated since external beam radiation and brachythearpy, endoscopic laser therapy or argon plasma coagulation, bougienage, and stent implantation are rival palliative procedures with comparable survival time and lower morbidity [4]. Endoscopic therapy is useful for patients with poor performance status, in whom other treatments have failed.

Ethanol-induced tumour necrosis (ETN) is simple, cheap and readily available. Uses of intratumoral injection of absolute alcohol for palliation of dysphagia have been reported. However, to the best of our knowledge, there have been no such reports from India. In India, the capital outlay needed for stenting or laser therapies is out of reach. The cost of an antireflux stent for palliation of carcinoma of the gastroesophageal junction is very high and was not available, but other methods of palliation of dysphagia for growth in the upper, middle, and lower third of oesophagus such as chemoradiotherapy, feeding jejunostomy, and stenting were available and were being used in our institute. Hence, we initiated a study to evaluate the effect of intratumoral injection of absolute alcohol in palliation of dysphagia due to carcinoma of the oesophagogastric junction and to observe the improvement in QOL.

## Methods

This was a prospective clinical interventional study conducted at the Jawaharlal Institute of Postgraduate Medical Education and Research (JIPMER) between September 2004 and June 2006. The study was approved by the Institute Ethics Committee. All patients with dysphagia due to unresectable malignant oesophageal strictures involving oesophagogastric junction, not suitable for other methods of palliation like chemoradiotherapy or non-availability of other methods of palliation like antireflux stents or laser or argon plasma, were included in the study. Informed consent was obtained from these patients. A detailed history was elicited regarding dysphagia, regurgitation of feeds, QOL, and other symptoms. Dysphagia was graded using Modified Takita’s functional grades of dysphagia [5]. QOL was assessed by modified European Organisation for Research and Treatment of Cancer (EORTC) QLQ C30 questionnaire [6]. This consists of 30 questions based on physical symptoms, emotional interactions, intellectual activity, economic independence, and self- perception of wellness. Each question was scored on a scale of 1 (‘very poor’) to 5 (‘very good’). Patients were assessed clinically and with Barium swallow, abdominal ultrasonogram, and chest X-ray. CT scan was done in nine patients. All patients were subjected to upper gastrointestinal endoscopy (UGIE) and biopsy was taken to confirm malignancy. Six patients underwent explorative laparotomy and were found to be inoperable.

UGIE was done by EPM-3400, PENTAX endoscope (PENTAX Corporation, Tokyo Japan) and the following parameters were noted: the site and length of lesion, gross appearance of tumour, and degree of circumferential involvement, stomach, and duodenum whenever possible.

After noting the findings, an endoscope was passed up to the point of obstruction. Six to ten cubic centimetres of absolute alcohol in 1 cc aliquots was injected circumferentially into the tumour at this point using disposable sclerosing needles (23G) manufactured by MTW endoskopie company. These needles were again reused after sterilisation. The patients were then kept under observation for the next 6 h, and a chest X-ray was then taken. If there was no evidence of perforation, patients were allowed to take a fluid diet and advised to start solids as tolerated, starting the next day. Any adverse effects of the injection were noted after each session. All patients were discharged, unless there were complications, with proper dietary advice.

Patients were followed up weekly. During each follow up, dysphagia grade and QOL score were noted, as mentioned earlier. UGIE was repeated and reinjections were performed if required. Reinjections were stopped after a maximum of four sessions if there was no improvement in dysphagia or if the patient developed complications. If the dysphagia improved, the injection sessions were continued until patients were able to swallow semisolid food (dysphagia grade 3). After this, patients were reviewed every four weeks or earlier, if dysphagia worsened. The dysphagia score and QOL score before and after alcohol injection, the number of sessions for the endoscope to enter into stomach and the number of sessions required for primary dysphagia relief, and the mean interval for the recurrence of dysphagia were analysed. Statistical analysis was done using Statistical Package for the Social Sciences version 13. A non-parametric test like Friedman Test and Wilcoxon Signed Ranks Test were used for comparing the dysphagia grade and QOL before and after absolute alcohol injection; *P* value <5% was considered significant.

## Results

**Figure 1.**
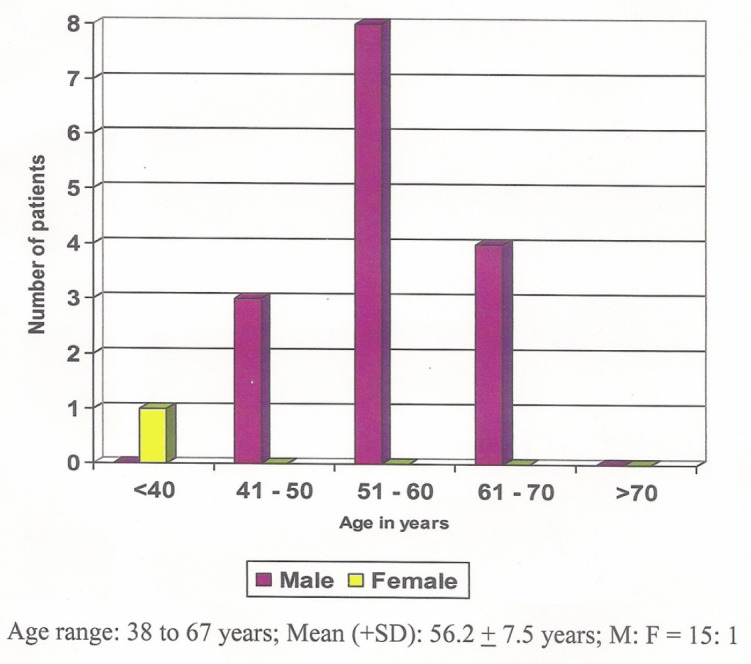
Age and sex distribution of the 16 patients with gastroesophageal junction carcinomas.

**Figure 2.**
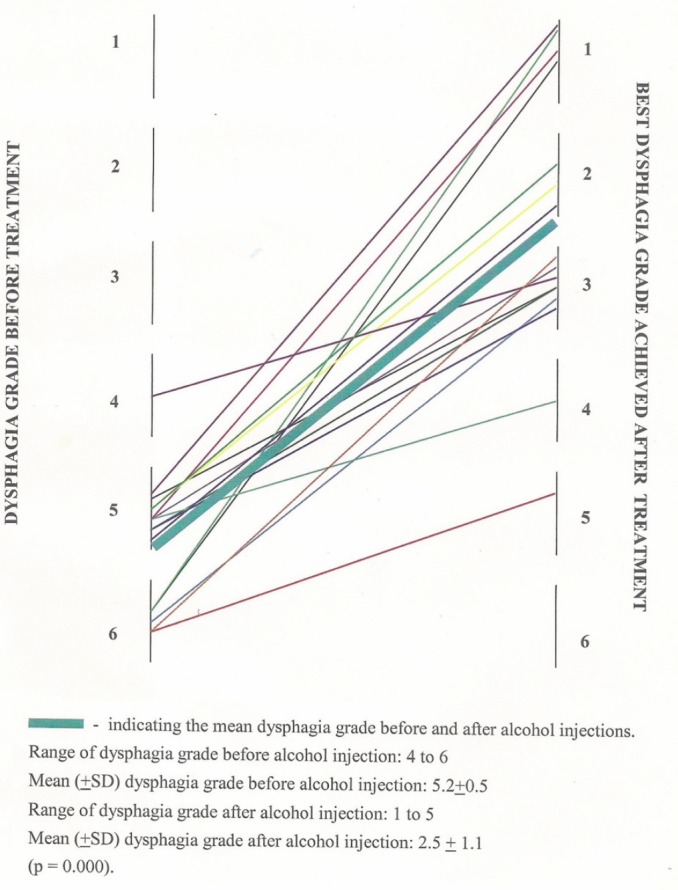
Dysphagia grade of 16 patients before treatment and the best grade achieved after alcohol.

A total of 16 consecutive patients with dysphagia due to unresectable malignant oesophageal strictures involving oesophagogastric junction were included in the study. The mean age of the 16 patients was 56.2 ± 7.5 years (Figure 1). Of the 16 patients, one was a female. Of these patients, 75% were in their sixth or seventh decades. Thirteen patients were diagnosed as having adenocarcinoma and the rest as squamous cell carcinoma. Prior to injection, 11 patients had grade 5 dysphagia; four patients had grade 6, and one patient had grade 4 dysphagia. The mean dysphagia grade was 5.2 ± 0.5 before alcohol injection. (Figure 2).

The total number of alcohol injection sessions ranged from one to seven, of which seven patients had two sessions. The mean number of sessions was 2.9 ± 1.7. Dysphagia relief was observed in 14 patients; the number of sessions required primarily for tolerating semisolid ranged from one three sessions with a mean of 1.3 ± 0.6 sessions. Dysphagia was not relieved in two patients, in one of whom, dysphagia was not relieved even after four sessions and the other died on the third day following the first injection. Following dysphagia relief, the patients were followed up, and once dysphagia worsened, reinjections were done. The number of reinjection sessions ranged from one to six with a mean of 1.7 ± 1.8.

The mean interval between the last primary injection sessions to the first secondary injection session, i.e., the time taken for the recurrence of dysphagia ranged from 14 to 180 days with a mean of 31.3 ± 18.6 days and with a median of 28 days. During the initial endoscopy, the endoscope could not traverse the growth in 15/16 patients. During the follow up period, among the 14 patients, the endoscope could pass across the stricture and enter the stomach in nine patients. There was correlation between endoscope traversing the stricture and improvement in dysphagia. The mean number of sessions required for the endoscope to traverse the stricture was 1.5 ± 1.0. The mean dysphagia score when the endoscope traversed the stricture was 3 ± 1.1.

During the follow up period, two patients had swallowing difficulties despite the clearance of luminal disease after the fourth session, but they were able to tolerate solids better than fluids and were able to swallow better in erect posture. The volume of alcohol injected per session was in the range of 3 to 10 cc and the mean volume of alcohol injected per session was 6.98 ± 1.8 cc.

**Figure 3.**
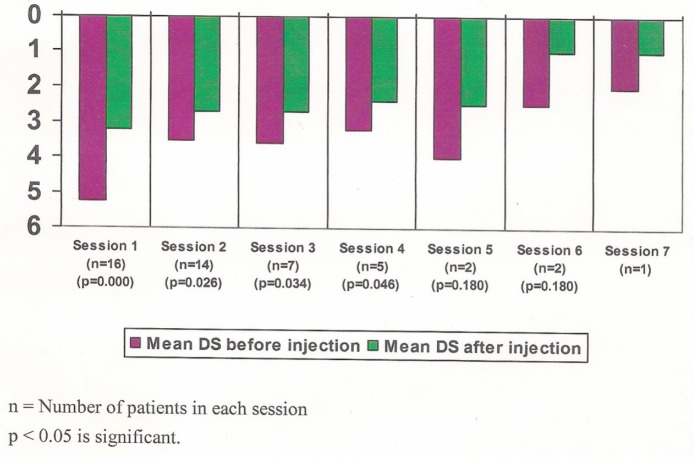
Mean dysphagia score before and after alcohol injection for each session.

The dysphagia grade before and the best grade after alcohol injection are shown in Figure 2. Following injections, the dysphagia grade, in seven patients improved to grade 3, in four patients to grade 1, and in three patients to grade 2. One patient following injection died on the third day and dysphagia grade was four, in another patient there was no improvement of dysphagia even after four sessions, though initially, following first session dysphagia grade improved form grade 6 to 5. There was statistically significant improvement in the mean dysphagia grade from 5.2 ± 0.5 to 2.5 ± 1.1 before and after alcohol injection, respectively (*p* < 0.0001). There was statistically significant improvement in the dysphagia before and after injections, in each session until the fourth session after which there was no further improvement (Figure 3).

**Figure 4.**
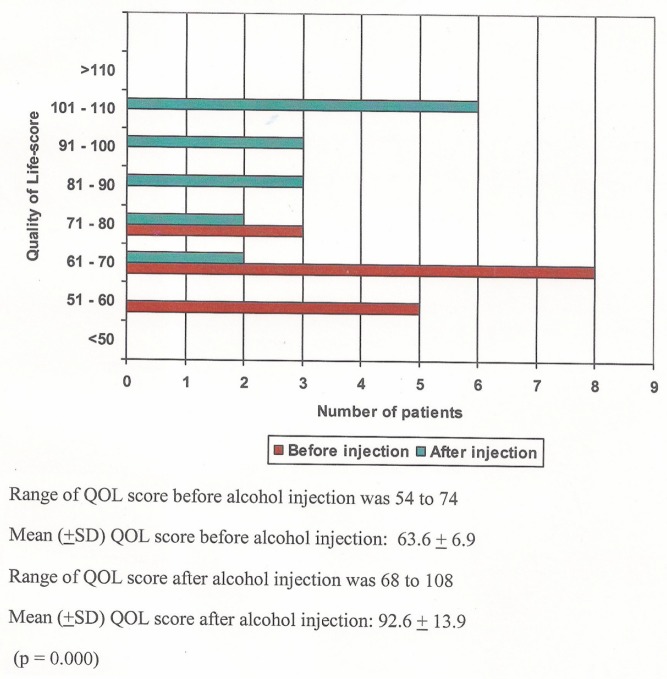
QOL score before and after alcohol injection.

All patients had QOL score between 50 and 75. QOL scores had improved following injection and score was between 90 and 110 in ten patients, between 70 to 90 in four patients, and in two patients it was 60 to 70. There was statistically significant improvement in the mean QOL score from 63.6 ± 6.9 to 92.6 ± 13 before and after alcohol injection, respectively (p < 0.0001) (Figure 4).

**Figure 5.**
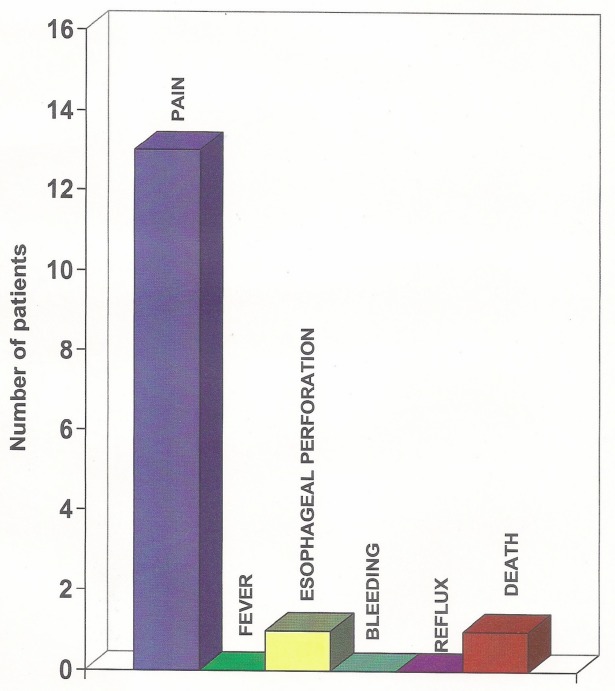
Morbidity and mortality.

Thirteen patients had chest pain during injections. None of them had fever, bleeding, or reflux from injections. One patient had oesophageal perforation which was diagnosed one week following third session of injection. One patient died on third day following first session of injection (Figure 5).

According to our institutional purchase price, the cost of the product required per session which included injection sclerotherapy needles which were reused after sterilisation, was Rs. 240/-. The survival period ranged from 3 to 220 days and the mean duration of survival was 84.3 ± 67.7 days. The dysphagia free survival ranged from 23 to 175 days with a mean of 71.2 ± 62.7 days.

## Discussion

In most patients with carcinoma of the oesophagus and gastric cardia, the disease is too advanced at presentation for there to be any prospect of cure [7]. Which therapy is chosen for the palliation of patients with unresectable oesophageal cancer depends on the symptoms requiring attention, the technology available, physician experience and training, and patient desires [8]. Identifying the most appropriate method of nutritional support for a patient with oesophageal cancer must be individualised [9].

Pilot studies of alcohol injection appeared in the literature approximately 20 years ago, and few uncontrolled series performed since then have documented high success rates. However, to the best of our knowledge, there has been no such study from our country. In India, a developing country, capital outlay needed for either stenting or laser therapies which are well established palliative techniques are out of reach in many centres. The patients in our study were assessed by a dedicated team of surgeons and radiotherapists and were found to be unfit for surgery and other modalities of palliation.

The mean age (56.2 ± 7.5 years) of the 16 patients was lower in our study when compared with other studies as in Nwokolo *et al* [10]. In Bown’s study where laser palliation was used, the mean age was 71.6 years [11]. In Sabharwal’s study where stenting was used, the age was 71.6 years [12]. However, when compared with Maroju’s study conducted in our institute where they had used stenting for palliation, the mean age was 57 ± 11 years and was similar to our study [13].

Prior to injection, the mean dysphagia grade was 5.2 ± 0.5, i.e., patients had difficulty in tolerating liquids. In Monga’s study where laser palliation was used, the mean dysphagia grade at presentation was 3.33 (Bowns grading) and was similar to our study [14]. It was also similar to other studies of ETN, as shown in Table 1. In Maroju study from our institute where they had used stenting for palliation and the presenting score ranged from 4 to 6 with a median of 4 (Takita’s scoring) [13] and was similar to our study. Following injections, the dysphagia grade had improved in 14 patients. Improvement in dysphagia was seen two to three days following injections. The improvement was similar to other studies of ETN, as shown in Table 1.

In our study, the number of sessions required for the relief of primary dysphagia ranged from one to three sessions with a mean of 1.3 sessions and median of one session. This was similar to Nwokolo’s study [10] where the number of sessions to reach the best dysphagia grade ranged from one to three with a median of one session. The time taken for the recurrence of dysphagia ranged from 14 to 80 days with a median of 28 days. This was similar to other studies of ETN, as shown in table above. During the secondary injection sessions, dysphagia improved with single session of alcohol injection. With self expanding metallic stents relief of dysphagia is immediate with a single session and patency is maintained unless complications develops [20]. Laser treatment usually require between two and four sessions to achieve initial dysphagia palliation, with further treatment every four to eight weeks following this [21]. The amount of alcohol injected per session was in the range of 3 to 10 cc and the mean volume of alcohol injected per session was 6.9 ± 1.8 cc and with a median of 7 cc. In the study conducted by Chung *et al*, the mean volume injected per session was 7.8 mL and in Nwokolo’s study it ranged from 1.5 to 29 cc with a median of 10 cc. In the study conducted by Chung *et al* [18] and Nwokolo *et al* [10], when the endoscope did not traverse the stricture, limited dilatation was done so as to permit the endoscope to traverse the stricture. If the lumen was totally occluded and the guide wire could not be passed, the proximal occluding part of the tumour was injected circumferentially. In Nwokolo’s study, the improvement in dysphagia in patients where dilatations was done prior to injection was similar to patients in whom only alcohol injections were done suggesting that the improvement was not because of dilatation. Carazzone *et al* in their study had one oesophageal perforation during the preliminary dilatation before the second session of alcohol injection [22].

In our study, during the initial endoscopy, the endoscope could not traverse the growth in 15/16 patients. Dilatations were not attempted in any of these patients, and alcohol was injected in the proximal occluding part of the tumour. In our study, there was significant improvement in mean dysphagia score following injection. During the follow up period among the 14 patients, the endoscope could pass across the stricture and enter into the stomach in nine patients. There was correlation between the endoscope traversing the stricture and patient improvement in dysphagia. Hence, we feel the injections can be done without dilatations, thereby avoiding the procedural complications related to dilatations like perforation.

During the follow up period, two patients had swallowing difficulties despite the clearance of luminal disease after fourth session but they were able to tolerate solids better than fluids and they were able to swallow better in erect posture. This might have resulted from motility disturbances secondary to muscle infiltration by tumour.

The ultimate goal of palliation is to improve the QOL. In our study, there was statistically significant improvement in the mean QOL score from 63.6 ± −6.9 to 92.6 ± 13.9 before and after alcohol injection, respectively. This parameter was not assessed in other alcohol injection studies. Our finding was similar to other studies which have demonstrated improved QOL in patients with inoperable oesophageal cancer following relief of dysphagia [23]. In the study conducted by Maroju Nanda Kishore *et al* in our institute where they had used stenting for palliation, Median QOL score was 72 (62–94) before stenting, increased to 107 (80–133) after the procedure [13]. Louis’s study, where laser was compared with intubation for palliation, showed QOL in both the groups had a negative correlation with the severity of dysphagia [7].

Thirteen patients had chest pain during injection; one patient had oesophageal perforation, this patient subsequently underwent feeding jejunostomy but got himself discharged against medical advice and was lost to follow up. There was no incidence of reflux in our study. One patient died on the third post injection day; though leak was suspected, it was not proven. The exact cause of death in this patient could not be established. Sudden cardiac death was another possibility thought of. Carazzone *et al*, in their study comparing ETN versus laser therapy, showed similar dysphagia improvement, and dysphagia-free intervals between treatments, but 78% of ETN patients experienced distressing levels of pain and one oesophageal perforation occurred during the preliminary dilatation before the second session of alcohol injection [22]. Chung *et al*, in their study, had complications that included mediastinitis in 1/36 patients and tracheoesophageal fistulas in 2/36 patients. There were no significant complications in other alcohol injection studies. With self-expandable metallic stents Song *et al* reported reflux in 9 (19%) out of 47 patients [20]. Non-refluxing stents have been employed to avoid these problems. Perforation accounts for the majority of significant early morbidity in laser treatments and occurs in 1–6% of patients [7].

In our study, the survival period after the first session of injection ranged from 3 to 220 days and the mean duration of survival was 84.3 ± 67.7 days. In Chung’s study [18], where alcohol injections were done the mean survival was 82 days. In Nwokolo’s study[10], the median survival after first session was 93 days. Louis *et al* showed that the mean survival in the group following laser therapy was 6.1 months and for the endoscopic intubation group was 5.1 months [7]. A recent randomised study by Dallal and colleagues comparing thermal ablative palliation with SEMS reported significantly longer survival in patients who underwent ablative therapies; 125 days versus 68 days, respectively (*p* < 0.005), with no explanation for this finding [24]. In a prospective randomised trial, Shah *et al* have reported improved survival, dysphagia, time between treatment, and time for stent requirement in those patients getting ETN and external beam radiation compared with those receiving only ETN [25]. By looking at the results of brachytherapy [26]. We have plans to compare ETN with endoluminal brachytherapy with ETN alone prospectively.

According to our institutional purchase price, the cost of the procedure per session which included the injection sclerotherapy needles which were reused after sterilisation was Rs. 240/-. If the needles had been used only once, then the cost per session would have been Rs. 1800/-. The cost of stenting using SEMS, works out to be Rs. 40,000/-.

A variety of approaches are available for the palliation of malignant dysphagia due to oesophageal cancer. Patients who are debilitated, poor surgical candidates or have recurrence of disease after surgery should be considered for endoscopic palliation [25–28]. Alcohol injection, laser therapy, photodynamic therapy, and argon plasma coagulation can be used primarily as debulking modalities to attempt reestablish oesophageal luminal patency [26].

In conclusion, endoscopic intramural injection of absolute alcohol significantly improves dysphagia and QOL. It requires no additional investment for most endoscopy units, is cheap, requires no hospitalisation, and is easy to perform. It provides effective palliation with complications comparable with other forms of palliation. If necessary, it can be repeated for more lasting palliations. It can be used for the oesophageal growth at any site as long as a reasonable view of the tumour is possible. With relief of dysphagia lasting a month, ETN has all the hallmarks of a good palliative technique.

## Conflicts of interest

The authors have no conflicts of interest to declare.

## Figures and Tables

**Table 1.** Studies on palliation of dysphagia by ethanol induced tumour necrosis.

Study	Total number of patients	Mean dysphagia score before injection	Mean dysphagia score after injection	Mean interval between treatments in days	Complications
Payne *et al* [15]	11	3	0.9	32	Nil
Fiorini *et al* [16]	19	3.22	1.47	45	Nil
Nwokolo *et al* [10]	32	3	1	28.5	Nil
Moreira *et al* [17]	9	3.4	1.2	31.5	Nil
Chung *et al* [18]	36	2.7	1.4	35	Mediastinitis-1 Tracheoesophageal fistula-2
Guitron *et al* [19]	6	3.6	0.8	–	Nil
In this paper	16	5.19^*^	2.50^*^	31.29	Oesophageal perforation-1

* Scoring according to Modified Takitas scoring; in other studies Bowns dysphagia scoring was used.
